# High‐resolution 3‐D scanning electron microscopy (SEM) images of DOT^TM^ polynucleotides (PN): Unique scaffold characteristics and potential applications in biomedicine

**DOI:** 10.1111/srt.13667

**Published:** 2024-04-01

**Authors:** Michael James Kim, Hyun‐Jun Park, Rae‐Jun Jung, Chee‐Youb Won, Seul‐Ong Ohk, Hong‐Taek Kim, Nark‐Kyung Roh, Kyu‐Ho Yi

**Affiliations:** ^1^ Aeon Medical and Aesthetic Centre Singapore Singapore; ^2^ Maylin Clinic (Chungdam) Seoul South Korea; ^3^ Pharmaresearch Co., Ltd. Integrated R&D Center Sungnam South Korea; ^4^ Leaders Aesthetic Laser and Cosmetic Surgery Center Seoul South Korea; ^5^ Maylin Clinic (Apgujeong) Seoul South Korea; ^6^ Division in Anatomy and Developmental Biology Department of Oral Biology Human Identification Research Institute BK21 FOUR Project Yonsei University College of Dentistry Seoul South Korea

**Keywords:** polydeoxyribonucleotide, Polynucleotide, scaffold, scanning electron microscopy

## Abstract

**Introduction:**

Polynucleotides (PN) are becoming more prominent in aesthetic medicine. However, the structural characteristics of PN have not been published and PN from different companies may have different structural characteristics. This study aimed to elucidate the structural attributes of DOT™ PN and distinguish differences with polydeoxyribonucleotides (PDRN) using high‐resolution scanning electron microscopy (SEM) imaging.

**Materials and methods:**

DOT™ PN was examined using a Quanta 3‐D field emission gun (FEG) Scanning Electron Microscope (SEM). Sample preparation involved cryogenic cooling, cleavage, etching, and metal coating to facilitate high‐resolution imaging. Cryo‐FIB/SEM techniques were employed for in‐depth structural analysis.

**Results:**

PDRN exhibited an amorphous structure without distinct features. In contrast, DOT™ PN displayed well‐defined polyhedral shapes with smooth, uniformly thick walls. These cells were empty, with diameters ranging from 3 to 8 micrometers, forming a seamless tessellation pattern.

**Discussion:**

DOT™ PN's distinct geometric tessellation design conforms to the principles of biotensegrity, providing both structural reinforcement and integrity. The presence of delicate partitions and vacant compartments hints at possible uses in the field of pharmaceutical delivery systems. Within the realms of beauty enhancement and regenerative medicine, DOT™ PN's capacity to bolster cell growth and tissue mending could potentially transform approaches to rejuvenation treatments. Its adaptability becomes apparent when considering its contributions to drug administration and surgical procedures.

**Conclusion:**

This study unveils the intricate structural scaffold features of DOT™ PN for the first time, setting it apart from PDRN and inspiring innovation in biomedicine and materials science. DOT™ PN's unique attributes open doors to potential applications across healthcare and beyond.

## 1 INTRODUCTION

The field of aesthetic medicine has witnessed a significant increase in the utilization of biostimulatory materials, including Polylactic Acid (PLLA), Calcium Hydroxylapatite (CaHA), Polydioxanone (PDO), Hyaluronic Acid (HA), Polydeoxyribonucleotide (PDRN), and Polynucleotide (PN).^1^ These materials come in various forms, ranging from absorbable threads that can be strategically inserted into the skin to powder forms that are reconstituted and injected as a liquid or gel directly into target areas. Absorbable threads, owing to their physical properties, can exert tensile forces on tissues, imparting a lifting effect and enhancing structural strength until they gradually dissolve.^2^ Synthetic biostimulators such as PLLA and CaHA have a long history of use in aesthetic medicine, supported by substantial published evidence demonstrating their ability to stimulate collagen and elastin production within the skin. Despite this progress, the exact mechanisms behind their action remain elusive, with documented evidence suggesting an involvement of inflammatory processes.^3^, ^4^

However, it's important to acknowledge certain drawbacks associated with these biostimulators. Firstly, many of them are not naturally occurring molecules in the human body and are instead synthetically manufactured. Additionally, the use of threads, while effective, introduces a higher level of invasiveness, potentially leading to complications such as infection, dimpling, scarring, or protrusion. Injectable biostimulators, on the other hand, carry the risk of severe complications like tissue necrosis and even blindness in cases of inadvertent intravascular injection. Delayed Inflammatory Reactions (DIR), hypersensitivity reactions, and granulomas are also documented risks. Unlike hyaluronic acid (HA), most injectable biostimulators are not reversible, making their use a careful decision.^5^ Even though HA injections can be reversible with hyaluronidase, this still does not exclude its use from developing side effects. Furthermore, while inflammation is increasingly recognized as a significant contributor to the aging process and delayed complications, the precise mechanisms through which inflammation adversely affects aging in the broader context of the aging process remain a largely uncharted territory, warranting further investigation.^6^

Polynucleotide (PN), a compound gaining increasing attention in the field of aesthetic medicine, deserves an introduction to its unique characteristics. One remarkable advantage of PN is its exemplary safety profile, with no reported cases of necrosis or blindness resulting from intravascular compromise to date.^5^ Despite this promising safety record, PN's mode of action had long been assumed to mirror that of polydeoxyribonucleotides (PDRN), even though no published evidence supported this assumption. Both PN and PDRN were considered A_2_A receptor agonists, believed to stimulate angiogenesis and growth factors, among other functions.^2^, ^7^

However, empirical observations in clinical practice began to reveal distinct differences in outcomes, with more pronounced improvements observed when PN was used for aesthetic and cosmetic indications.^7^

This prompted a critical question: What sets PN apart from PDRN beyond differences in size and molecular weight? If their modes of action are ostensibly the same, why opt for PN, which can be more painful and less cost‐efficient for patients?

Notably, multiple companies worldwide manufacture PN, but specific details about their PN formulations remain largely undisclosed to the public. In the case of DOT™ PN (Rejuran^®^, PharmaResearch Ltd, Republic of Korea), its uniqueness lies in the proprietary technology, DOT™, employed during its production process. The base DOT™ PN molecules are extracted from testes cells of wild chum salmon (*Oncorhynchus keta*), harvested during their return to the river for spawning. Intriguingly, a decade ago, internal scanning electron microscopy (SEM) imagery of DOT™ PN revealed an irregular scaffold‐like structure with few discernible patterns or significant features. However, this enigmatic visual offered the potential to unlock the key differences between PN and PDRN, shedding light on how PN could play a distinct role in the treatment of various aesthetic/cosmetic indications. Consequently, to unveil the physical structure of DOT™ PN and differentiate it from PDRN, SEM imagery of both DOT™ PDRN and DOT™ PN was undertaken, paving the way for further insights into their divergent actions and clinical applications.

## 2 METHODS AND MATERIALS

In this study, an investigation into the structural characteristics of Polynucleotides (PN) (Rejuran^®^), specifically focusing on samples obtained from PharmaResearch Ltd. (Republic of Korea) was conducted. To facilitate this examination, a Quanta 3‐D field emission gun (FEG) scanning electron microscope (SEM), manufactured by the Field Electron and Ion (FEI) Company (USA), was employed. The sample preparation process was conducted ensuring the integrity of the specimen for imaging. This process involved several crucial steps:

Firstly, a precisely measured aliquot of 100 µL of the PN specimen was immobilized onto the SEM/FIB holder. To enable high‐resolution imaging, cryogenic cooling techniques were employed. Liquid nitrogen was introduced for a brief duration, rapidly lowering the temperature to well below its boiling point (−196°C). Subsequently, the specimen was swiftly immersed in this ultra‐cold environment. The prepared specimen was then transferred to a specialized preparation chamber meticulously maintained under vacuum conditions.

At a controlled temperature of −145°C, a delicate cleavage of the specimen was executed using a knife. Following cleavage, the temperature was maintained at approximately −95°C for a duration of 5–10 min to facilitate etching.

To enhance imaging capabilities, the specimen's surface was coated with a thin layer of Au/Pd metal. This metal coating process was carried out using a located target within the preparation chamber. The prepared specimen was then seamlessly transferred to the SEM/FIB chamber, which featured a cryo stage designed for cryogenic imaging.

Subsequently, in‐depth image analysis was conducted to elucidate the structural characteristics of the PN specimen. Cryo‐FIB/SEM imaging experiments were conducted using the Quanta 3D FEG system, coupled with an Alto 2500 cryo‐transfer system supplied by Gatan, Inc. (United Kingdom).

The imaging process entailed several critical steps: Cu‐deposited polymer substrates were securely attached to a copper stub using carbon tape. Sessile droplets were sprayed onto the prepared substrates, which were then rapidly submerged into a liquid nitrogen slush within 0.5 s. Approximately 1 s later, the freezing chamber was efficiently evacuated to maintain the desired cryogenic conditions. The prepared samples were then swiftly transferred into a specialized preparation chamber, pre‐ and maintained within a temperature range of approximately −120°C to −140°C.

Metal deposition on the samples was achieved through plasma sputtering, employing a 3‐mA current for a duration of 60 s. The metal‐coated samples were seamlessly transferred into a dedicated microscope chamber, also pre‐evacuated to a pressure of 10^−5^ mbar and precooled to a temperature within the range of −120°C to 140°C. Cryo‐SEM images were acquired using a 5‐keV electron beam of energy and an electron current of 11.8 pA. Focused Ion Beam (FIB) milling was conducted using a 30‐keV gallium ion beam and an ion current of 1 nA. For EDS spectra collection, the electron beam energy was increased to 15 keV.

## 3 RESULTS

**FIGURE 1:**
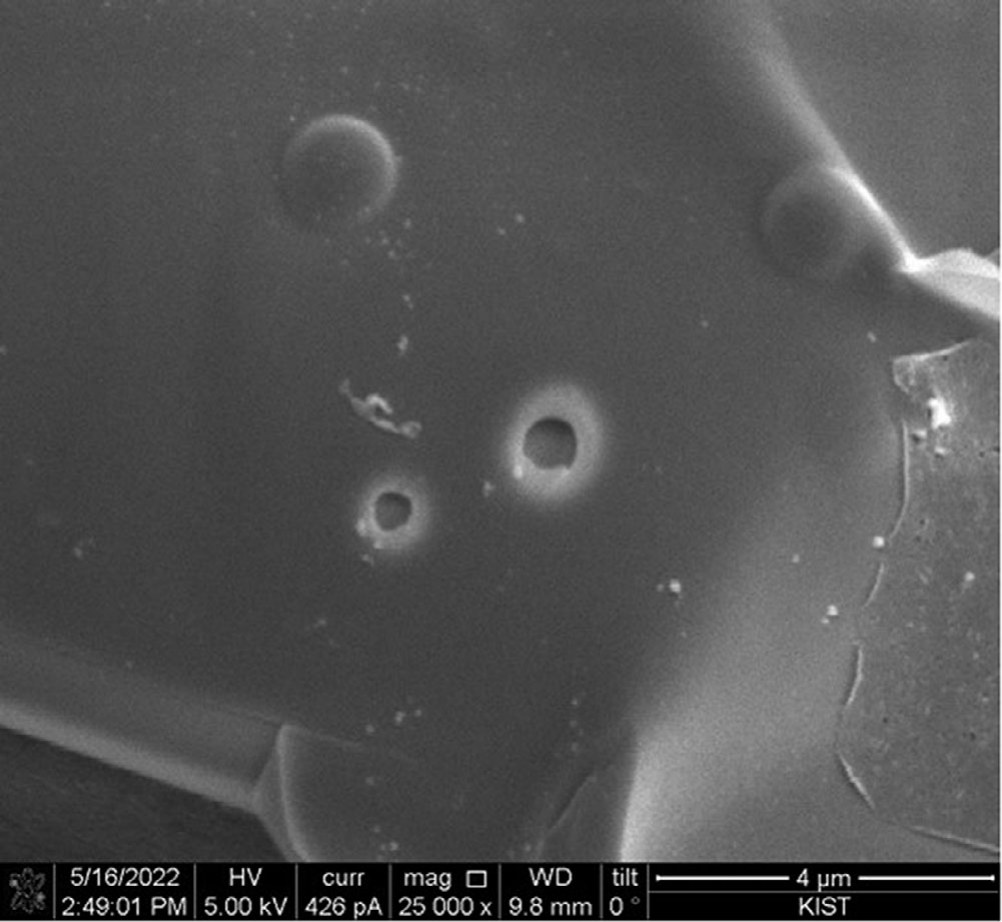
DOT PDRN (×25,000 magnification) [Quanta 3D FEG (Field Electron and Ion Company, USA)].

**FIGURE 2:**
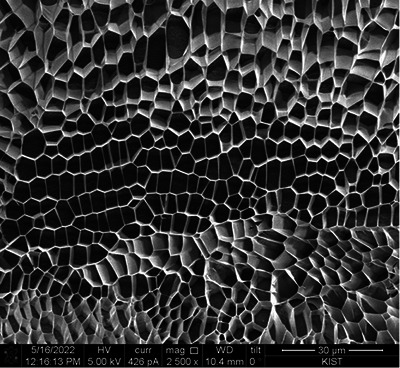
DOT Polynucleotide (×2500 magnification) [Quanta 3D FEG (Field Electron and Ion Company, USA)].

**FIGURE 3:**
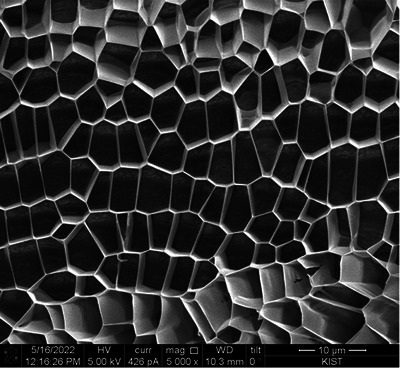
DOT Polynucleotide (×5000 magnification) [Quanta 3D FEG (Field Electron and Ion Company, USA)].

**FIGURE 4:**
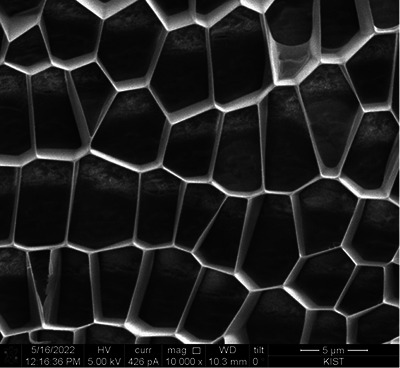
DOT Polynucleotide (×10000 magnification) [Quanta 3D FEG (Field Electron and Ion Company, USA)].

In Figure 1, DOT™ PDRN is depicted, revealing a structure that lacks distinct architectural features, appearing amorphous and undefined. Figures 2, 3, 4 offer a comprehensive view of DOT™ PN's unique characteristics. Notably, DOT™PN exhibits a well‐defined structural well‐defined wall pattern, forming polyhedral shapes reminiscent of cellular structures. These polyhedral cells present smooth and uniform thickness walls, measuring less than 0.1 micrometers in thickness. The cells appear empty, devoid of any identifiable contents. Moreover, their sizes vary, with an average diameter ranging from approximately 3 to 8 micrometers, as evident in Figure 4. The multi‐directional alignment of these cell walls in cross‐sections is attributed to the gel‐like nature of the sample, which lends itself to fluidity. During the freezing process, the cells assume diverse alignments. Many of the spaces within DOT™ PN take on hexagonal or pentagonal configurations, although some variations exist. A remarkable feature is the seamless tessellation, with no discernible empty spaces between the cells. Despite the presentation of these observations in cross‐sections, a closer examination of cells in the upper and lower regions of Figure 2 reveals that the walls enclose three‐dimensional spaces within the structure.

## 4 DISCUSSION

PN have become more popular in aesthetic/cosmetic applications around the world. It is highly biocompatible and an ingredient that is derived from chum salmon or trout gonads therefore differentiating itself from other biostimulators which are synthetic polymer‐based products. DOT™ PN differ from DOT™ PDRN in that: (1) PN are extracted from testes whereas PDRN are extracted from sperm cells, (2) PN have longer nucleotide chains (3) PN have a higher molecular weight and as seen in this study (4) PN has a scaffold structure absent in PDRN.

However, in terms of research and published articles, the number of PN‐related papers is much lower than those on the topic of PDRN. PDRN has been studied and published extensively. From 2016 to 2020, there were approximately 609 published articles related to PDRN and 70 were reviewed by Kim et al.^8^ A Pubmed search done independently found approximately 57 more PDRN related papers from 2021 to 2023 September.

As early as 1989, Bruroni et al. used PDRN in cervical ectropion patients^9^ and in 1990, Perino et al. used PDRN for post‐cauterization re‐epithelialization.^10^ Muratore et al.^11^ in 1997 used human placental PDRN on human knee skin fibroblasts in primary culture and in 1999, Thellung et al. published an important article on the involvement of A2A receptors in the mechanism of action for PDRN.^12^ There have been additional publications on PDRN and its involvement in skin graft donor site healing,^13^, ^14^ promotion of corneal fibroblasts in culture,^15^, ^16^ human osteoblast proliferation,^17^, ^18^ and even angiogenesis.^19^, ^20^

One unique finding is that PDRN promoted cyclobutene pyrimidine dimer (CPD) repair in UVB‐exposed dermal fibroblasts.^21^ Recent discoveries showed PDRN and its properties in anti‐melanogenesis,^22^, ^23^ anti‐allodynic,^24^ mitochondrial biogenesis,^22^ and even fat browning for potential anti‐obesity applications.^25^

On the other hand, PN are longer chains of nucleotides which have a much less robust number of research articles. Park KY et al. in 2016 showed that DOT™ PN improved pore size, skin thickness, skin tone, melanin, wrinkles and sagging in five patients.^26^

Kim JH et al. in 2018 showed the beneficial effect of DOT™ PN for improvement of post‐thyroidectomy scars in a randomized, double‐blinded, controlled trial of 44 patients. Those treated with PN had significantly improved Vancouver Scar Scale (VSS), 3‐D analysis of height, patient satisfaction and erythema index.^27^

Lee YJ et al. in 2022 performed a randomized, double‐blind split face trial with DOT™ PN and non‐cross‐linked hyaluronic acid filler injection in 27 subjects. The improvement rates of pore volume and roughness were higher in the PN group but the improvement was not significant for global aesthetic improvement scale, visual analog scale or dermal density.^28^

In more recent developments, Kim MJ et al. published two cases where DOT™ PN was successfully used to volumize areas in the face traditionally only treated with other dermal fillers.^5^ Lee at al. surveyed 557 Korean physicians and found that a considerable majority of doctors used PN for a variety of facial erythema with more than 80% finding it “effective” or “highly effective.”^29^

Important to note that in all of the above studies, (1) there were no serious adverse events reported (safety) and (2) there is no objective data on the mechanism of action. It is hypothesized that due to the similarity of PN and PDRN molecular structure, that PN will have its mechanism of action similar to PDRN. However, up to date, this has not been scientifically demonstrated.

Also, as far as the authors are aware, there are no peer‐reviewed publications presenting the physical images of PN. This can be significant because one of the main differences between PDRN and PN has been known to be the presence of a “scaffold‐like” structure in PN since the inception of DOT™ PN about 9 years ago. If this is a main differing factor between the two molecules, then that “scaffold” structure might have the key to unlocking the Polynucleotide mechanism of action which has yet to be elucidated.

The results uncovered in this study provide deeper insights into the structural intricacies of DOT™ PN and how it sets itself apart from DOT™ PDRN. These revelations carry substantial implications (in addition to above mentioned mechanism of action) across multiple domains, spanning from aesthetic applications (rejuvenation), musculoskeletal regeneration, wound healing, angiogenesis, anti‐aging, bioengineering, stem cell differentiation, delivery mechanisms, and even in the realm of biotensegrity.^9^, ^10^, ^11^

As the mechanism(s) of PN have not yet been elucidated yet, starting out with PN morphology can be one of the starting points of investigation. The data which is available from the SEM imagery in this study is limited and only some basic observations are possible. However, through these observations, the authors hope that further research and investigations will follow soon.

1 Shape

Examination of images reveal that almost all of the “cells” or “shapes” of DOT™ PN are 5‐sided pentagons with the majority being 6‐sided hexagons. The size and shape of all the cells are slightly different and this can be due to the gel‐like consistency of the product. This is in stark contrast to other biostimulator polymers which have spherical or irregularly shaped particles.

Hexagons are seen in nature in various places. As bubbles reduce their surface tension and minimize surface area, they form hexagonal arrays. The spatial arrangement of atoms in diamonds, graphite and snowflakes are also hexagonal. This happens in nature due to the balancing of force vectors to achieve the most energetically efficient configurations.^30^

In living tissues, hexagons in groups of cells can be seen as a balance between tensional forces on the internal of cells and those from the surroundings of the cells.^31^, ^32^

The hexagonal arrangement can also be seen in the arrangement of actin and myosin fibers in muscle in the urothelial surface, the microstructure of the avian lung, eyes of insects and of course the honeycomb of the bee.^30^

The significance of polymer shape of implants was studied in 1976 by Matlaga et al. and there have been other authors discussing the effect of geometric shape on scaffold structures.^33^, ^34^, ^35^

Hayashi et al. mentions that a scaffold with hexagonal cells is superior in both porosity and mechanical strength. Also, in terms of cell responses, hexagonal channels are superior to triangular and square shaped channels. Although the study was done in relation to bone regeneration, they concluded that the shape of the scaffold yields greater effect than the surface area of each scaffold. Thus, the shape difference at the single scaffold level (microscopic) affects the whole system (macroscopic).^33^

2 Size

The length of the walls of the cells ranges from approximately 1–7 µm but the majority seem to be in the 4–6 µm range. The diameter of the cells also is approximately 3–5 µm. Evans et al. described the approximate diameter of fibroblasts to be 28 µm.^36^

The DOT™ PN scaffold structure is multiple times smaller than the size of an average fibroblasts which can allow more binding of the fibroblasts to the scaffold walls.

According to the “receptor saturation” model proposed in 1987 by Dembo and Bell,^37^ the number and density of receptor adhesive sites between the cell (integrin binding sites) and substrate (ligand binding sites) will affect the fibroblast activity. Whether the size of the DOT PN scaffold is too small, too large or within the optimal range for fibroblast binding is still to be determined.

Varani et al. showed that in older skin, extracellular matrix (ECM) collagen is more fragmented and shorter leading to less attachment points for fibroblasts leading to shrinking of the cells, loss of mechanical stimulation and eventually less production of collagen.^38^, ^39^

Injection of DOT™ PN can possibly enhance the matrix scaffold in the ECM allowing fibroblasts to attach and regain functionality. Fisher et al. demonstrated that small, rounded fibroblasts placed in constrained matrixes displayed spread morphology and restored their mechanical properties.^40^

3 Alignment/Tessellation

One characteristic of DOT™ PN in the SEM images is that the cells are intricately connected and arranged in a repeated pattern without gaps, that is, in tessellation. From a 2‐D perspective, there are only three regular polygons which can achieve tessellation: triangles, squares or hexagons. Among these three, the hexagon has the most efficient perimeter to area ratio. Of course, in the case of DOT™ PN, the walls of the cells are not equal length but still tessellate the surface.

However, the product and its effect in vivo must be assessed from a 3‐D perspective. The Wearie‐Phelan structure is a model to solve the problem of filling space with no gaps with cells of equal volume and minimum surface area. In other words, the most efficient form of tessellation of space. The walls of this structure are composed of pentagons and hexagons.^41^ Further investigation is needed, but the pentagonal and hexagonal cell shapes of DOT™ PN as seen in the SEM images of this study closely resemble the theoretical pentagonal and hexagonal shapes of the Wearie‐Phelan structure walls.^41^ More in‐depth analysis of the 3‐D structure of DOT™ PN can lead to understanding more about its mechanical properties and role in vivo, especially the ECM.

The observations of DOT™ PN's polyhedral tessellation structure, as vividly depicted in the electron microscopy images, hint at a level of organization and interconnectedness that evokes the elegant architecture found in living organisms. The uniform thickness of its walls and the presence of empty cells within the structure bear resemblance to the intricate cellular organization observed in nature.

Apart from the documented applications regarding tissue regeneration, this DOT™ PN scaffold/lattice structure can be applied to stem cell research, bioengineering and as a delivery vehicle.

1 Bioengineering

Douglas et al. in 2009 demonstrated that DNA can be used to build nano‐structures with precise control.^42^

In 2023, Michelson et al. published research on how DNA molecules can be used as a nano‐lattice due to its superior strength to weight ratios. When combined with other materials like silica, they can produce a lightweight and high‐strength framework. Since DNA assembly can produce diverse lattice types, this approach can lead to more extensive material developments with varying mechanical responses.^43^

Gasperini et al. also showed that DNA crystal films formed through self‐assembly can be possibly used to deter UV radiation and promote wound healing when applied on the skin.^44^

2 Stem cell differentiation

Keeping in mind that DOT™ PN can modulate the elasticity of the ECM through its scaffold structure, it can be considered for future use in stem cell research and applications in the future, whether it may be in vivo or in vitro. Engler et al. suggest that “precommitting” stem cells to a specific lineage via appropriate in vitro matrix conditions can in part overcome an “inappropriate” in vivo microenvironment, emphasizing the importance of matrix/scaffold in determining the stem cell lineage specification.^45^

In 2013, Watt et al. explored how the stiffness of a substrate can affect how cells respond and differentiate to these differing ECM cues.^46^

It has also been demonstrated by Teo et al. that the alignment and size of nanogratings affected the differentiation of stem cells toward certain lineages without andy biomechanical factors. Therefore, different ECM compositions can direct stem cell differentiation. They found that cells are able to sense differences between micro and nanometer size gratings in addition to being more elongated on patterned nanogratings compared to “unpatterned” gratings.^17^

3 Delivery Vehicle

Delving deeper into DOT™ PN's characteristics, its thin walls and polyhedral shapes open intriguing avenues for innovative delivery mechanisms. The presence of empty cells within the structure presents the prospect of these cells acting as reservoirs for encapsulated substances. This, in turn, paves the way for controlled and sustained release, a critical factor in the development of advanced drug delivery systems. The applications are diverse, spanning both oral and injectable drug delivery, where precise control over dosage and release kinetics is paramount.

Combining bioactive molecules with other scaffolds for bone tissue repair and bone regeneration have already been published with promising results.^4^, ^17^, ^47^

Basic requirements for scaffolds have been discussed and have various viewpoints on what constitutes an “ideal” scaffold. Depending on the application, the criteria of course will vary. Lu et al. in 2012 listed the requirements for a skin scaffold to: (1) have low antigenicity (2) be able to promote efficient and fast cell adhesion and proliferation (3) have high mechanical strength and (4) to have open and interconnected porous architecture to allow cell penetration resulting in a uniform, homogenous cell distribution and tissue formation.^4^

Although discussed in the context of bone scaffolds, Li et al. explained that scaffolds are made to mimic the nanofobrous collagen ECM and thus provide a 3D environment for cells and tissue to grow on. They need to allow cell ingrowth by being highly porous and at the same time provide efficient transport of growth factors, oxygen, nutrients, and waste products. For larger scaffolds, it must also allow adequate vascularization to avoid necrosis at the core.^48^, ^49^

In 2023, Krishani et al. additionally stated that a scaffold should have a biodegradation rate proportional to the rate of new tissue formation and exit the body without interfering with other tissues and organs. They also mentioned that the scaffold should be highly biocompatible with negligible chronic immune responses, lasting no more than 2 weeks.^50^, ^51^, ^52^

Also, the scaffold should possess similar mechanical properties as the tissue it is being implanted into. Tensile testing and compressive testing are the conventional methods that need to be done to further evaluate the compatibility of a scaffold for its intended purpose.^52^

From this perspective, materials used in regenerative medicine for cosmetic purposes can have different characteristics and applications depending on the scaffold properties.

Lastly, DOT™ PNs tessellated and interlocking structural pattern can be applied to the principles of biotensegrity. Biotensegrity is a concept rooted in the tensional integrity of biological systems, underscores the significance of continuous tension and compression elements in maintaining structural stability. It also is a concept and theory connecting biology and physics that centers on how physical forces (i.e., from a scaffold) regulate cellular biochemical responses in a process known as mechanotransduction.^53^

Although R. Buckminster Fuller was known to have coined the term “tensegrity,”^53^ Professor Donald Ingber has further expanded the concept into biology, that is, Biotensegrity.^53^, ^54^, ^55^, ^56^, ^57^, ^58^, ^59^

## 5 CONCLUSION

These collective findings illuminate the unique structural attributes of DOT™ PN, setting it apart from DOT™ PDRN and unveiling its intricate polyhedral tessellation pattern. This structural distinctiveness holds significant promise for a wide array of potential applications across various scientific and medical domains, making DOT™ PN a subject of great interest for further research and exploration

In conclusion, the structural features of DOT™ PN, as unveiled in this study, inspire innovation across diverse fields, ranging from biomedicine to materials science. These findings mark the beginning of an exciting journey toward harnessing DOT™ PN's unique attributes for the betterment of healthcare and beyond.

## CONFLICT OF INTEREST STATEMENT

M.J.K, H.J.P. and N.K.R. are advisory board members of Pharmaresearch Ltd.

## Data Availability

Data are available on request to corresponding author.
